# Periodontitis associated with prostate cancer and other urological cancers in patients over 50 years of age: a cross-sectional study

**DOI:** 10.4317/medoral.27179

**Published:** 2025-08-16

**Authors:** Andrea Alexandra Ajalcriña-Martensen, Carlos Alberto Minchón-Medina, Juan Astigueta-Perez, Willy Gustavo Gamboa-Vicente, Luis Jason Ganoza-Larrea, Angel Steven Asmat-Abanto

**Affiliations:** 1 Orcid ID: 0009-0009-4473-8609. Student of Stomatology Study Program, Antenor Orrego Private University. Trujillo, Peru; 2 Orcid ID: 0000-0002-2441-5302. Professor of the Faculty of Physical Sciences and Mathematics, Department of Statistics, National University of Trujillo, Peru; 3 Orcid ID: 0000-0001-5984-3270. Professor of Human Medicine Study Program, Antenor Orrego Private University; 4 Orcid ID: 0000-0002-2356-9940. Medical surgeon, Urology Specialist, Assistant at Belén Hospital, and Undergraduate Professor of Human Medicine, Antenor Orrego Private University, Trujillo, Peru; 5 Orcid ID: 0000-0003-0807-7814. Master's Degree in Stomatology, Specialist in Periodontics and Implantology. Professor of Stomatology Study Program, Antenor Orrego Private University, Trujillo, Peru; 6 Orcid ID: 0000-0001-5726-6692. Doctor in Stomatology, Specialist in Periodontics. Research Professor of Human Medicine Study Program, Antenor Orrego Private University, Research Professor of Stomatology Study Program, Antenor Orrego Private University, Trujillo, Peru

## Abstract

**Background:**

Periodontitis triggers systemic inflammatory reactions that may be associated with different oncological diseases, such as prostate cancer (PC), although little information exists on the possibility of this association. To determine whether periodontitis is associated with PC and other non-metastatic urological cancers in patients over 50.

**Material and Methods:**

This cross-sectional study was conducted between August and September 2024 at the outpatient clinic of the Belén de Trujillo Hospital (HBT) and the Regional Institute of Neoplastic Diseases “Dr. Luis Pinillos Ganoza” - IREN Norte (IREN), in Trujillo, Peru. In total, 192 patients were evaluated: 64 with PC, 64 with non-prostatic urological cancer (NPUC), and 64 with non-oncological urological disease (NOUD). Reliability was determined through inter- and intra-evaluator calibration to diagnose periodontitis, gingivitis inflammation, and plaque control. The corresponding specialist physician diagnosed PC, NPUC, and NOUD. Statistical analysis was performed using the Chi-square and Kruskal-Wallis tests, including nonparametric multiple comparison tests, with a significance level of *p*<0.05.

**Results:**

An association between periodontitis and PC was found in patients over 50. PC was also directly associated with moderate periodontitis (*p*=0.014) and generalized gingivitis inflammation (*p*=0.019). Concerning NPUC, an association was found with periodontal pockets of 3.5 - 5.5 mm (*p*=0.031) and last visit to the dentist more than one year ago (*p*=0.012).

**Conclusions:**

There was an association between periodontitis and PC in patients over 50. Patients with moderate periodontitis and generalized GI were more likely to present PC compared with patients with NOUD. In addition, there was a greater probability of presenting PC versus NPUC in patients with periodontal pockets of 3.5-5.5 mm and in those whose last visit to the dentist was more than one year ago.

** Key words:**Periodontitis, prostatic neoplasms, prostate cancer, urologic neoplasm, periodontal diseases, gingivitis, periodontal pockets.

## Introduction

Periodontitis is a multifactorial chronic inflammatory disease associated with dysbiosis (1-3), characterized by the continuous destruction of dental supporting tissue (2). Its prevalence worldwide ranges from 45% to 90%, while the severe form affects between 11.2% and 20% of people (4).

Moreover, it is one of the leading causes of tooth loss in adults (1) and negatively affects the quality of life of people who suffer from it (5).

Urological cancers account for a quarter of all existing cancers; the most common of these are bladder, kidney and prostate cancer (PC) (6). PC is ranked the second most prevalent cancer in men and one of the leading causes of death worldwide (7). Approximately 95% of PC cases correspond to adenocarcinomas of acinar origin, with the average age for diagnosis being 66 years (8,9). Its main risk factors include age, race, prostatitis, prostate-specific antigen (PSA) level, and family history (10,11).

Periodontitis compromises the body's first line of defense by triggering reactions that can impact systemic health (12,13). It has been hypothesized that periodontitis and PC are related by periodontal pathogens and proinflammatory mediators (cytokines and chemokines) (13,14). Gram-negative bacteria such as *Treponema denticola* and *Porphyromonas *gingivalis** have been found in prostatic secretions and the dental biofilm of patients suffering from periodontitis and prostatitis (15). Furthermore, research studies have suggested that *Porphyromonas *gingivalis** and *Fusobacterium nucleatum* may alter cancer patients' conditions through various mechanisms (15-17).

PC is the most common cancer in men in over half of the world, specifically in 112 out of 185 countries (18). In 2020, over 1.4 million new cases of this cancer were recorded, making it a significant public health problem. Although there are studies on the possible association between periodontitis and PC, their results have been contradictory. For this reason, the present study was conducted to determine whether periodontitis is associated with PC and other non-prostatic urological cancers in patients over 50 years of age, including some possible intervening factors, such as sociodemographic data, oral health status, and comorbidities. The knowledge generated in this study contributes to a better understanding of the relationship between oral health and cancer for the benefit of the scientific community and patients in dentistry and oncology. Moreover, it invites researchers to create hypotheses for future work that will contribute to reducing the global incidence of cancer and improving the quality of life of cancer patients.

## Material and Methods

This cross-sectional study was conducted at HBT and IREN, Trujillo (Peru), between August and September 2024. The sample consisted of 192 subjects: 64 patients diagnosed with PC, 64 with NPUC, and 64 with NOUD being evaluated. This sample size was calculated using the formula for pairwise comparison of groups, using data generated by a pilot study with 20 patients per group and with the following parameters: n (Total sample size between the two groups compared), r=1 (Proportion of a group of interest compared with the control), Zα/2=1.96(Normal value with type I error of α=5%), Zβ=1.645(Normal value with type II error of β=5%, power of 95%), ΠA=0.63375 (Relative effect of periodontitis in patients with prostate cancer compared to patients without cancer), M=4 (Number of degrees of severity of periodontitis: No periodontitis, mild periodontitis, moderate periodontitis and severe periodontitis) and ) y ρi (Mean proportion of patients in the group of degree of periodontitis severity between the groups being compared, estimated with a pilot sample). The selection method was non-probabilistic for convenience.

The patients included in this study were male adults over 50 who attended the outpatient clinic with a histological diagnosis of PC or NPUC (with extension studies negative for metastasis) from the IREN or NOUD from the HBT. These two hospitals were chosen because the patients diagnosed with cancer at the HBT are referred for care at IREN; since both hospitals belong to the Ministry of Health of Peru, the sociodemographic factors tend to be homogeneous. Those excluded from the study were patients receiving hormonal or bisphosphonate treatment, taking drugs causing gingivitis enlargement, having fewer than six teeth, with underlying immunosuppression problems, and those who did not agree to participate in the research.

The present study was approved by the Faculty of Human Medicine (Res. Nro. 2609-2024-FMEHU-UPAO) and the Bioethics Committee of the Universidad Privada Antenor Orrego (Res. Bioethics Committee No. 01219-2024-UPAO), of the Ethics and Research Committee of the Hospital Belén de Trujillo (ID CIEI - HBT/114-2024) and of the Institutional Committee on Research Ethics IREN NORTE (No. 082-2024-IREN NORTE-DG-CIEI). The above-mentioned organizations observe strict compliance with the principles established in the Declaration of Helsinki adopted by the World Medical Association and the General Health Law of Peru No. 26842.

Before being asked to participate, all patients received information about the purpose of the research. Upon acceptance, they were given the informed consent to read and sign. The specialist physician of the corresponding service made the diagnosis of PC and NPUC through biopsies and corresponding complementary tests. Subsequently, the lead author assessed the presence of periodontitis according to the CDC/AAP classification validated and agreed upon by the Centers for Disease Control and Prevention and the American Academy of Periodontology, using a North Carolina probe and taking into account the following criteria: no periodontitis, mild periodontitis (≥2 interproximal sites with CAL ≥3 mm and 2 interproximal sites with PPD ≥4 mm, not on the same tooth, or ≥1 site with PPD ≥5 mm), moderate periodontitis (≥2 interproximal sites with CAL ≥4 mm, not on the same tooth, or ≥2 interproximal sites with PPD ≥5 mm, not on the same tooth), and severe periodontitis (≥2 interproximal sites with CAL ≥6 mm, not on the same tooth, and ≥ 1 interproximal site with PPD ≥5 mm). The results were recorded in the corresponding data collection form, which collected basic demographic and covariate information.

The reliability of the method for measuring periodontitis was determined by evaluating 20 patients, undergoing intra- and inter-rater calibration after receiving prior training, by the principal investigator and a professor specialist in Periodontology from the Stomatology Study Program at the Universidad Privada Antenor Orrego (Trujillo, Peru).

Considering that the CDC/AAP epidemiological index requires evaluation of the level of clinical attachment and probing depth, intraclass correlation was used to evaluate the distal, middle and mesial areas of the vestibular surface of the teeth of a complete sextant per patient.

Calibration was also performed to determine the presence of dental biofilm and bleeding on probing. The intra-rater intraclass correlation for probing depth in the distal, middle, and mesial zones was estimated at 0.973, 0.916, and 0.865, respectively. For attachment loss, it was 0.945, 0.863, and 0.875 for the distal, middle, and mesial zones, respectively. For the presence of plaque and bleeding on probing, the values were 0.986 and 0.984, respectively. Relative to the inter-rater intraclass correlation (principal investigator-specialist), from the distal to mesial regions, the values were 0.976, 0.907, and 0.851 for probing depth; and 0.961, 0.899, and 0.861 for clinical attachment level, respectively. Likewise, for the presence of plaque and bleeding on probing, the correlations were 0.980 and 0.990, respectively.

The data collected were processed automatically using the statistical program IBM SPSS version 29 (IBM, Armonk, NY, USA). The results are presented in Tables showing the comparative frequencies of the three groups of patients and in-ring charts. Group comparisons were performed using the Chi-square test for homogeneity, Kruskal-Wallis, and non-parametric multiple comparison tests. Periodontitis, sociodemographic factors, oral and periodontal health status, and comorbidities were associated with PC when using binary logistic regression analysis, in which PC is the event of interest, and, in the first case, the complementary event was NOUD. In the second case, the complementary event was NPUC. Likewise, the relationship between the variables under study, according to the covariates, was evaluated using binary logistic regression. The tests' significance level was considered if *p*<0.05.

## Results

**Figure 1:**
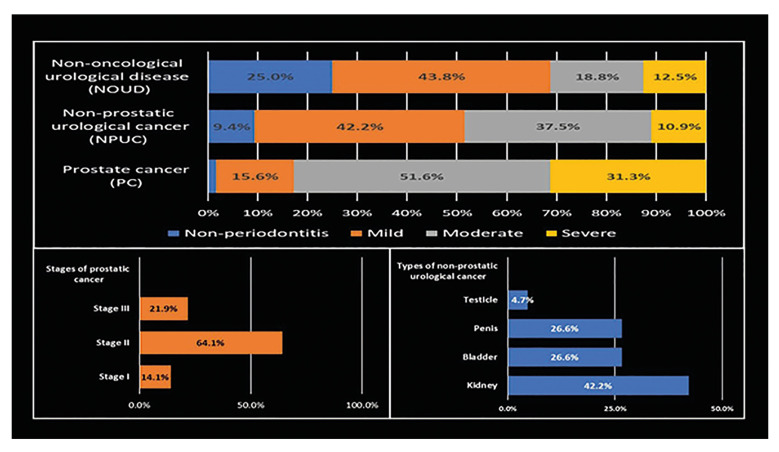
Periodontitis and urological cancer characterization at Belén de Trujillo Hospital and the Regional Institute of Neoplastic Diseases “Dr. Luis Pinillos Ganoza”, in Trujillo, Peru. August - September, 2024.

In total, 192 male patients over 50 and a mean age of 66.4 years (+/- 8.7) were evaluated. They were divided into the three groups as mentioned above, and the following results were obtained:

As may be observed in Fig. 1, periodontitis was present in 75% of patients with NOUD, 98.4% in patients with PC, and 90.6% in patients with NPUC. Severe periodontitis was present in 12.5%, 31.3%, and 10.9% of the abovementioned groups. Furthermore, men with PC were predominantly in stage II (64.1%) and kidney cancer occurred most frequently (42.2%) among NPUC.

Due to the small number of patients without periodontitis, they were grouped with those who presented mild periodontitis, forming the category “without periodontitis/mild periodontitis” for statistical purposes.

When the prevalence of periodontitis was compared between the groups studied, differences were found, as seen in Table 1 (*p*=0.000 for Chi-square and Kruskal-Wallis tests). Furthermore, when the multiple comparison test was used, it was determined that men with PC presented more severe levels of periodontitis than those in the NOUD (*p*=0.000) and NPUC (*p*=0.000) groups. There was no difference between these latter two groups (*p*=0.162).

Table 2 compares the homogeneity of the study groups to associated factors. Relative to the sociodemographic characteristics, differences were found between age groups (*p*=0.002), family background (*p*=0.011), and educational level (*p*=0.044). Concerning oral health, the groups showed differences in gingivitis inflammation (GI) (*p*=0.000), presence of periodontal pockets (*p*=0.000), plaque control (*p*=0.000), and last visit to the dentist (*p*=0.031). Moreover, relative to comorbidities, the groups only showed differences concerning Type 2 diabetes mellitus (DM2) (*p*=0.000).

Table 3 shows the binary logistic regression analysis used to associate the factors under study with PC compared to NOUD. A greater tendency to present PC was found in men with moderate periodontitis (*p*=0.014) when compared with the category without periodontitis/mild periodontitis. This was also the case in those who presented generalized GI (*p*=0.019) compared to those who showed gingival health. On the contrary, unexpectedly, a lower tendency to present PC was found in those who did not brush their teeth (*p*=0.009) when compared with those who did so two or more times a day, and in those who presented DM2 (*p*=0.000) compared to those who did not show it.

In Table 4, binary logistic regression analysis was also used to associate the factors under study with PC when compared with NPUC. No difference was found in the tendency to develop PC in relation to periodontitis, but a higher frequency of PC was found in patients with periodontal pockets of 3.5-5.5 mm (*p*=0.031) when compared with those who did not have periodontal pockets and when their last visit to the dentist was more than 12 months ago (*p*=0.012).

## Discussion

Periodontitis releases inflammatory mediators, which, together with pathogenic microorganisms, can cause infections and inflammation (19), leading to the beginning of tumor conditions in various organs, including the prostate (20).

Over half of the NOUD patients and almost all of the PC and NPUC patients at IREN suffered from periodontitis. This could be because cancer patients are prone to a weakened immune system due to cancer treatments (21). Since periodontitis is a chronic inflammatory disease that directly impacts the immune system, it would cause the deregulation of the oral microbiome in these patients, thus complicating their general health status and worsening their prognosis and quality of life (22).

At IREN, the patients with PC were predominantly in stage II. This may be because, over the last decade, studies for early detection of this pathology, such as prostate-specific antigen (PSA) analysis and multiparametric magnetic resonance imaging, have evolved (23). The types of NPUC most frequently found at IREN corresponded to kidney cancer, followed by penile and bladder cancer, coinciding with the findings of Michaud *et al*. (24), who found that the most frequent NPUC were kidney and bladder cancers. In Peru, the incidence of urological cancers has increased, mainly in the rate of mortality due to kidney cancer, a trend that disproportionately affects men (25). In this country, as in other South American countries, access to health services can be limited by geography, low budgets allocated to the health sector, political corruption, and high patient demand. This prevents the early detection of urological neoplasms and other oncological diseases (25).

The categories without periodontitis and mild periodontitis were grouped for statistical analysis, considering that mild periodontitis has a lower CAL and PPD than moderate and severe periodontitis. Deep periodontal pockets, found mainly in these last two categories, contain specific microorganisms, such as *Porphyromonas *gingivalis** and *Treponema denticola*, which could trigger a proinflammatory host response with cytokines capable of producing direct carcinogens (2,26).

Patients with PC had more severe levels of periodontitis than the NPUC and NOUD groups, coinciding with the findings of Lee *et al*. (27), who indicated that patients with PC predominantly presented severe periodontitis. There was no agreement with Michaud *et al*. (24), who stated that there was a strong association of advanced periodontitis with kidney and bladder cancer, however, not with PC, but in patients who had never smoked. The influence of periodontitis on PC is mediated by chronic systemic inflammation and shared risk factors, such as impaired immune function, genetic susceptibility, and the presence of oral pathogenic microorganisms in the genitourinary system (22).

Differences were found between the groups studied according to age, family history, educational level, GI and probing depth, presence of dental plaque, DM2, and last visit to the dentist. This is why it was decided to perform the multivariate analysis. It was possible to establish the association independently of each factor studied using this statistical technique. Significant differences were found in the degree of GI between cancer and non-cancer patients, which could indicate an altered immune system (21) and the critical impact that persistent chronic inflammation could have on oncogenic activity. Moreover, patients with PC presented periodontal pockets more frequently than the other study groups. These ecosystems are niches that harbor bacteria that trigger cell proliferation, production of interleukins and matrix metalloproteinases (MMPs), closely related to increased systemic inflammation, predisposing patients to a higher risk of cancer or related complications (26).

Most of the cancer patients evaluated had not been to the dentist for longer than a year, a worrying situation that should lead to developing strategies to improve oral-periodontal health education, and a periodontal service must be implemented to support the multidisciplinary treatment of cancer patients. Considering the citation by Chen *et al*. (28), who indicated that periodontitis was a modifiable risk factor for PC and that an effective treatment could reduce the risk.

Binary logistic regression analysis reinforced the finding that PC was associated with greater severity of periodontitis and generalized GI. This was consistent with the findings of Lee *et al*. (27), as previously mentioned, possibly because periodontal infections and persistent inflammation cause a systemic inflammatory reaction. This condition is known to contribute to the development of cancer. In contrast, unexpectedly, a lower likelihood of developing PC was found in those who did not brush their teeth compared to those who brushed twice or more per day and among those with DM2 when compared with those who did not brush. This disagreed with Kim *et al*. (21), who indicated that PC was related to DM2 due to chronic inflammation and oxidative stress. The atypical results relative to tooth brushing and DM2 in this study may be due to the small number of patients with controlled DM2, so the suggestion is to expand this in future studies. One possible explanation for brushing frequency is that patients who brush more frequently may respond to pre-existing oral health problems, such as periodontitis, associated with an increased risk for PC. Likewise, no more significant probability of showing PC was found in patients with periodontitis than in the NPUC group. In this group, however, a higher likelihood of presenting NPUC was observed in patients with periodontal pockets of 3.5-5.5 mm and in those whose last visit to the dentist was more than a year ago. These findings are consistent with those of Michaud *et al*. (29), who indicated that most patients with PC had at least one site with attachment loss of 3 mm or more, and one-third had attachment loss of 6 mm or more. Lack of tooth brushing and poor dental visits increase the prevalence of severe periodontitis. Therefore, it is necessary to reinforce health education and periodic periodontal check-ups, especially for oncological patients.

The cross-sectional design of the present study limited the capacity to evaluate the temporal relationships between the variables. Therefore, the suggestion is that longitudinal studies should be conducted to obtain additional information, validate these results, and explore possible causal relationships in studies with larger sample sizes. In addition, in the present study, a group of patients with NPUC was assessed; however, this group is quite heterogeneous concerning cancer type, so the results should be viewed cautiously, as this heterogeneity does not allow definitive conclusions to be drawn, only exploratory data.

Considering the finding of periodontal pockets in patients with urological cancer, frequent periodontal evaluation should be incorporated into clinical guidelines for oncological care. Periodic dental assessment is recommended for the early detection and treatment of periodontal pockets since the eventual increase in depth will require surgical therapies (30). This will complicate both health needs and budgets. Health authorities are called upon to propose and create new guidelines and care protocols for these patients.

## Figures and Tables

**Table 1:** Comparison of levels of periodontitis in patients with NOUD, PC and NPUC.

Periodontitis	NOUD		PC		NPUC		X^2^	p
	N°	%	N°	%	N°	%		
No/mild	44	68.8	11	17.2	33	51.6	37.874	0.000
Moderate	12	18.8	33	51.6	24	37.5		
Severe	8	12.5	20	31.3	7	10.9		
Total	64	100	64	100	64	100		
Kruskal-Wallis	p =0.000						-	-
Multiple comparisons	A		B		A		-	-

**Table 2:** Comparison of levels of periodontitis in patients with NOUD, PC and NPUC.

Factors			NOUD		PC		NPUC		X^2^	p
			N°	%	N°	%	N°	%		
Sociodemographic data	Age group	50-60	21	32.8	7	10.9	23	35.9	21.264	0.002
		61-70	21	32.8	25	39.1	28	43.8		
		71-80	18	28.1	22	34.4	12	18.8		
		> 80	4	6.3	10	15.6	1	1.6		
	Family history of prostate cancer	With	13	20.3	27	42.2	27	42.2	8.987	0.011
		Without	51	79.7	37	57.8	37	57.8		
	Level of studies	No studies/Primary	29	45.3	20	31.3	15	23.4	9.769	0.044
		Secondary	18	28.1	24	37.5	33	51.6		
		Higher	17	26.6	20	31.3	16	25.0		
	Socioeconomic level	Class A-D	19	29.7	27	42.2	21	32.8	2.384	0.304
		Class E	45	70.3	37	57.8	43	67.2		
	Marital status	Married	24	37.5	12	18.8	20	31.3	5.695	0.223
		Single	34	53.1	45	70.3	38	59.4		
		Widowed/Divorced	6	9.4	7	10.9	6	9.4		
Periodontal health status	Gingival inflammation	Healthy gingiva	25	39.1	1	1.6	6	9.4	82.302	0.000
		Localized inflammation	29	45.3	18	28.1	48	75.0		
		Generalized inflammation	10	15.6	45	70.3	10	15.6		
	Presence of periodontal pockets	No periodontal pockets	25	39.1	2	3.1	22	34.4	29.465	0.000
		Periodontal pocket of 3.5-5.5mm	29	45.3	43	67.2	36	56.3		
		Periodontal pocket > 5.5 mm	10	15.6	19	29.7	6	9.4		
	Dental plaque	Adequate plaque control	34	53.1	9	14.1	9	14.1	32.967	0.000
		Inadequate plaque control	30	46.9	55	85.9	55	85.9		
	Brushing frequency	Does not brush	11	17.2	11	17.2	4	6.3	5.335	0.255
		Brush 1 time/day	21	32.8	25	39.1	29	45.3		
		Brush at least 2 times/day	32	50.0	28	43.8	31	48.4		
	Last visit to the dentist	≤ 12 months	12	18.8	25	39.1	16	25.0	6.933	0.031
		> 12 months	52	81.3	39	60.9	48	75.0		
Comorbidities	Smoking	Does not smoke	41	64.1	44	68.8	39	60.9	1.350	0.853
		Ex-smoker	20	31.3	17	26.6	20	31.3		
		Smoker	3	4.7	3	4.7	5	7.8		
	Alcoholism	Does not consume alcohol	29	45.3	38	59.4	36	56.3	5.969	0.201
		Ex consumer	24	37.5	20	31.3	15	23.4		
		Consumes alcohol	11	17.2	6	9.4	13	20.3		
	DM2	Presents	53	82.8	15	23.4	11	17.2	69.341	0.000
		Does not present	11	17.2	49	76.6	53	82.8		
	Obesity (body mass index)	Insufficient/Normal	30	46.9	38	59.4	31	48.4	4.123	0.390
		Overweight	29	45.3	20	31.3	24	37.5		
		Obesity	5	7.8	6	9.4	9	14.1		

**Table 3:** Association of factors in patients with PC compared with patients with NOUD.

Factors		Coefficient	Standard error	Wald Test	P	OR
Periodontitis	Moderate	3.365	1.367	6.063	0.014	28.944
	Severe	3.066	1.905	2.590	0.108	21.446
Age group	61-70	1.679	1.041	2.599	0.107	5.360
	71-80	2.176	1.157	3.535	0.060	8.814
	> 80	2.831	1.511	3.513	0.061	16.970
With a family history of prostate cancer		0.647	0.879	0.542	0.462	1.910
Level of studies	No studies/Primary	-1.804	1.007	3.213	0.073	0.165
	Secondary	-1.181	0.985	1.438	0.230	0.307
Gingival inflammation	Localized inflammation	2.214	1.391	2.535	0.111	9.156
	Generalized inflammation	4.212	1.796	5.500	0.019	67.514
Brushing frequency	Does not brush	-4.041	1.537	6.908	0.009	0.018
	Brush 1 time/day	-2.359	1.231	3.670	0.055	0.095
Last visit to the dentist > 12 months		-0.999	0.805	1.541	0.214	0.368
Presents DM2	Presents DM2	-3.407	0.880	15.003	0.000	0.033
	Constant	-1.253	1.773	0.499	0.480	0.286

**Table 4:** Association of factors in patients with PC compared with patients without NPUC.

Factors		Coefficient	Standard Error	Wald Test	P	OR
Periodontitis	Moderate	-0.098	0.870	0.013	0.910	0.906
	Severe	-1.861	2.000	0.866	0.352	0.156
Age group	61-70	1.012	0.721	1.974	0.160	2.752
	71-80	1.285	0.807	2.537	0.111	3.616
	> 80	2.560	1.324	3.735	0.053	12.932
With a family history of prostate cancer		0.192	0.529	0.132	0.716	1.212
Level of studies	No studies/Primary	-0.026	0.747	0.001	0.972	0.974
	Secondary	-0.480	0.655	0.537	0.464	0.619
Gingival inflammation	Localized inflammation	-1.138	1.603	0.504	0.478	0.320
	Generalized inflammation	1.772	1.817	0.951	0.329	5.884
Presence of periodontal pockets	Periodontal pocket 3.5-5.5mm	2.611	1.208	4.671	0.031	13.618
	Periodontal pocket > 5.5 mm	3.064	1.995	2.358	0.125	21.419
Brushing frequency	Does not brush	-0.920	0.868	1.124	0.289	0.398
	Brush 1 time/day	-0.776	0.971	0.639	0.424	0.460
Last visit to dentist > 12 months		1.663	0.661	6.326	0.012	5.276
Presents DM2	Presents DM2	0.083	0.710	0.014	0.907	1.087
	Constant	-2.590	1.594	2.639	0.104	0.075
